# Break and enter the retroflexed side branch when lock pick fails

**DOI:** 10.1093/ehjcr/ytae573

**Published:** 2024-10-23

**Authors:** Pitt O Lim

**Affiliations:** Consultant Cardiologist, Department of Cardiology, Atkinson-Morley Wing, St George’s University Hospitals NHS Foundation Trust, Blackshaw Road, Tooting, London SW17 0QT, UK

Side branch (SB) access can be very challenging. If the take-off is reverse-angled, coupled with ostial and proximal disease, wiring will be nigh impossible. Yamaguchi *et al.*^1^ discussed the ‘hairpin’ reverse-wiring technique but settled for a microcatheter looped 360° clockwise within a post-stenotic dilatation to provide the angulation and support needed to wire a SB, christened the ‘U-turn’ wiring method. This belatedly adds to the toolbox of bifurcation percutaneous coronary intervention (PCI). There are three additional points relevant to this topic. First, in Case 1, the wire inadvertently curved round-the-clock within an aneurysmal SB, dissected this vessel and entered main branch (MB). Proximal SB was stented, and it was stent-trapped behind MB stent, which was placed afterwards. Despite bifurcation kissing dilations, SB ostium recoiled significantly and MB stent at SB origin angiographically looked underexpanded—a fluoroscopic stent boost would have proven this point. Nevertheless, this experience was applied to Case 2, this time the wire was sheathed within a microcatheter. Second, an angled-tipped or dual-lumen microcatheter can facilitate SB access, and the ‘balloon-bounced’ approach; where a balloon is inflated in SB to redirect the wire into MB, or vice versa. Third, complex PCI operators nowadays are quite content modifying plaque within subintimal space and intentionally causing dissection; the so-called grenadoplasty and investment procedure, if all else fails.^2–4^

*Figure 1* illustrates a 75-year-old man who had coronary artery bypass grafting when he was 42 (1984)—40 years ago, by a famed cardiac surgeon who described the Ross procedure. He had left internal mammary artery grafted to diagonal artery, saphenous vein grafts to left anterior descending (LAD) artery, intermediate (INT) artery, and right posterior descending artery (RPDA). He had a diagnostic coronary angiogram when he was 65, his grafts to INT and RPDA were occluded, and an attempt was made to PCI the ungrafted circumflex (Cx) artery at a centre where the cardiologist who implanted the first coronary stent in the world worked. He had become more symptomatic despite medical therapy. Complex PCI to left main (LM)/INT/Cx bifurcation was therefore undertaken. The native right coronary artery was occluded, but distally, it was well collateralized from the grafted LAD. He had normal cardiac function on echocardiogram. The Cx was severely diseased with a ‘zigzag’ take-off, making it inaccessible to wiring. It was dilated across with a cutting balloon and a drug-coated balloon (DCB) was inflated from LM into INT, intended as an investment stagewise procedure. As a general rule-of-thumb, the dissected SB that occludes during PCI, if not stented across, recanalizes spontaneously in time—the trade-off is a small infarct which is unavoidable, unless collateralized. And DCB appears to facilitate follow-up PCI.^5^ More recently, SB subintimal tracking and re-entry has become routine amongst the complex PCI operators, making losing a SB ‘seemingly a thing of the past’.^6^ But at the expense of the occasional coronary perforation, as the skill set is acquired. In this case, fortuitously, Cx ostium flung open and the procedure was completed with bifurcation double-dilate crush stenting.^7^ He remains well over the past seven and a half years, now aged nearly 83.

**Figure 1:**
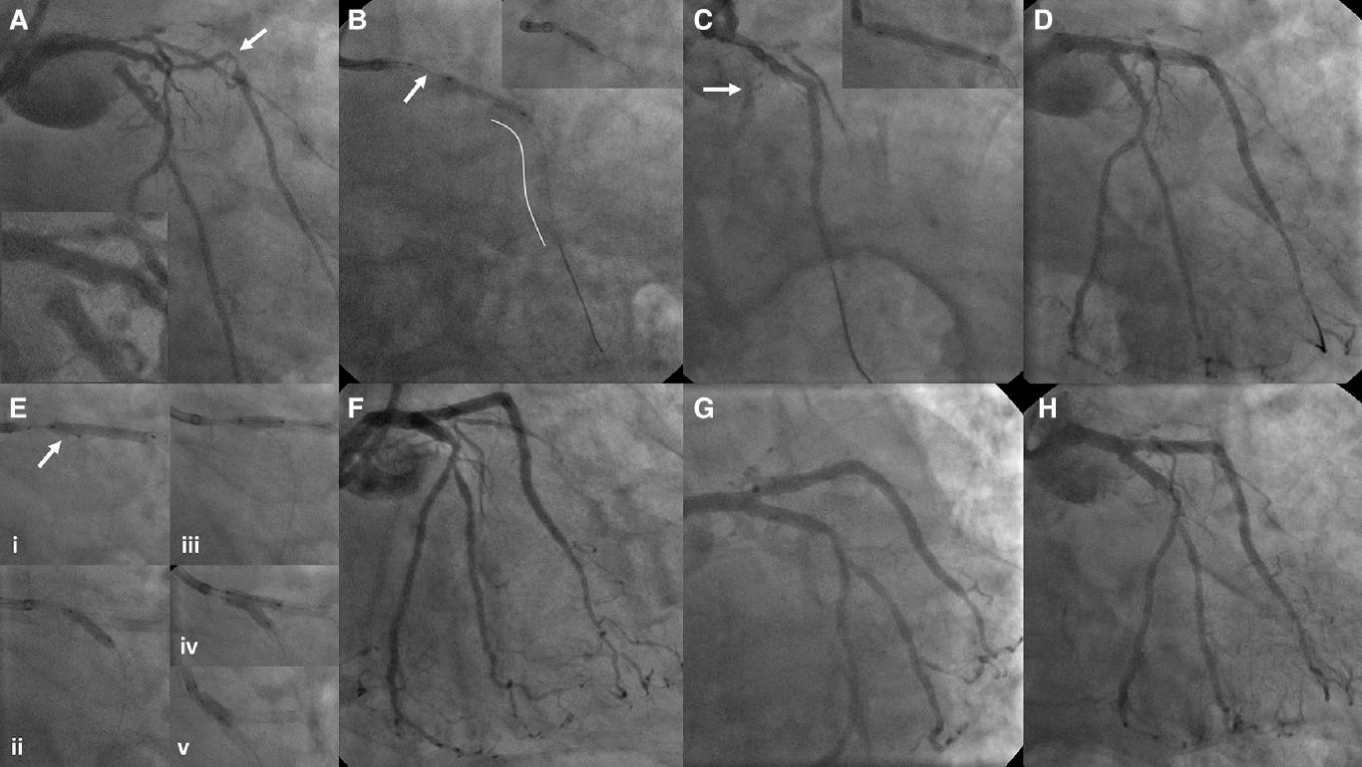
(*A*) The coronary angiogram shows a ‘tented’ or ‘pulled’ intermediate artery (arrow) where a saphenous graft is inserted, now occluded, and circumflex artery with a ‘Z’ ostial take-off, magnified in the inset. (*B*) Mid intermediate artery was stented with overlapping 2.5 × 26 and 2.25 × 18 mm drug-eluting stents (DES) (white line) covering the graft insertion site. A balloon was placed just shy off circumflex artery ostium, to bounce a hydrophilic wire (arrow) with various shapes into circumflex artery, but without success. Left main into intermediate artery across circumflex artery was dilated with a 3.0 × 10 mm cutting balloon (inset). (*C*) Following cutting balloon dilations, flow down circumflex artery was compromised (arrow). Left main into intermediate artery across circumflex artery was treated with a 3.0 × 30 mm drug-coated balloon (DCB), inflated to 16 atmospheres for 1 min (inset). (*D*) Following DCB deflation and withdrawal, type C dissection was noted in unstented proximal intermediate artery, but there was now free flow through the newly ‘sprung open’ circumflex artery ostium. (*E*) The ‘subintimal plaque modified’ circumflex artery was easily wired. (i) A 2.5 × 26 mm DES was deployed across left main into intermediate artery, overlapping with prior stents, with an uninflated ‘crush’ balloon straddling left main and circumflex artery (arrow). (ii) Following removal of the stent balloon, the parked balloon was inflated, crushing the proximal stent in the left main. (iii) The intermediate artery with ostially crushed stent was recrossed and dilated (first dilation). (iv) A 3.5 × 26 mm stent was deployed from left main into circumflex artery across intermediate artery ostium. The stent-trapped intermediate artery was recrossed and dilated (second dilation), and kissing balloon inflations undertaken in bifurcation, followed by proximal optimization in left main (v). (*F–H*) Final coronary angiograms in right anterior oblique 15°, spider and caudal views, respectively.

**Consent**: Obtained for the figure.

**Funding:** None.

## Data Availability

The data underlying this article are available upon reasonable request.
